# Structures of pentatricopeptide repeat proteins

**DOI:** 10.1107/S2053230X26002311

**Published:** 2026-03-30

**Authors:** Anuradha Pullakhandam, Crystal Cooper, Ian D. Small, Charles S. Bond

**Affiliations:** a School of Molecular Sciences The University of Western Australia Crawley Western Australia Australia; b Center for Microscopy, Characterization and Analysis The University of Western Australia Crawley Western Australia Australia; c Australian Research Council Centre of Excellence in Plants for Space Australia; University of York, United Kingdom

**Keywords:** pentatricopeptide repeat proteins, RNA recognition, protein crystallography, organellar gene expression, synthetic biology

## Abstract

Pentatricopeptide repeat (PPR) proteins constitute the largest family of RNA-binding proteins in land plants, playing crucial roles in organellar gene expression through sequence-specific RNA recognition. Including the first PPR crystal structure in 2013, 30 PPR and 83 ribosomal PPR crystal or cryo-EM structures have been deposited in the Protein Data Bank, encompassing both native and designer proteins in apo and RNA-bound states. This comprehensive survey catalogues all PPR crystal structures, analysing their crystallographic properties, structural features and contributions to understanding PPR function. Key insights include the elucidation of the PPR recognition code, the characterization of dramatic conformational changes upon RNA binding and the structural basis for the modular architecture that enables programmable RNA targeting. Designer PPR structures have validated and extended principles derived from native proteins, demonstrating their utility as synthetic biology tools. This structural catalogue provides crystallographers and plant biologists with a comprehensive resource for understanding PPR protein architecture and function.

## Abstract 2

A number of crystal, cryo-EM and solution structures of pentatricopeptide repeat proteins have been solved, revealing details of how they interact with single-stranded RNA and other proteins, as well as their conformational repertoire.

## 1. Introduction

Helical repeat proteins are modular proteins that are composed of tandem repeats that form an elongated, solenoid structure. These proteins consist of 20–40 amino acids per motif and form a solenoid structure with 2–30 repeats. Each motif is arranged in antiparallel helices (helices A and B) that are stacked to form a superhelical structure with concave and convex surfaces, which enable the effective binding of proteins or nucleic acids (Groves & Barford, 1999 ▸). The relative orientation of the motifs, the selectivity of residues, the repeat number and the overall curvature of these proteins play a key role in modular recognition, through which these proteins carry out a vast variety of functions such as transport, signalling, transcription and translation (Rubinson & Eichman, 2012 ▸; Barkan & Small, 2014 ▸; D’Andrea & Regan, 2003 ▸; Hammani *et al.*, 2014 ▸).

Comparative studies suggest that helical repeat proteins have expanded in number in eukaryotes through gene duplication and genetic recombination (Sharma & Pandey, 2016 ▸). For example, HEAT and armadillo repeats have a similar helical architecture with superimposable grooves and a conserved hydrophobic core, enabling protein–protein interactions and demonstrating a likely common ancestor (Andrade *et al.*, 2001 ▸). Tetratricopeptide repeat (TPR) proteins form a right-handed superhelical structure with concave grooves that enable them to bind ligands (Sharma & Pandey, 2016 ▸; Ponting *et al.*, 1999 ▸). Pentatricopeptide repeat (PPR) proteins are generally considered to have diverged from TPR proteins. In this evolutionary context, the structural relationship between TPR and PPR proteins has been thoroughly described (Gully, Shah *et al.*, 2015 ▸; Barik, 2021 ▸), highlighting the substantial similarities in sequence conservation and superhelical architecture. These similarities are remarkable given that the key conserved residues in PPR proteins enable nucleotide binding rather than peptide or protein binding (Small & Peeters, 2000 ▸), thus making them interesting candidates for programmable RNA-binding platforms.

PPR proteins represent the most abundant class of RNA-binding proteins in land plants, with over 450 members in *Arabidopsis thaliana* alone (Lurin *et al.*, 2004 ▸). These nucleus-encoded proteins are targeted to chloroplasts and mitochondria, where they regulate virtually every aspect of organellar gene expression, including RNA stability, splicing, editing, cleavage and translation (Barkan & Small, 2014 ▸). The defining feature of PPR proteins is their modular architecture, composed of tandem repeats of a degenerate 35-amino-acid motif that forms a characteristic helix–turn–helix structure.

The importance of PPR proteins in plant biology, combined with their potential as programmable RNA-binding tools, has driven intensive structural studies since 2013 (Yin *et al.*, 2013 ▸; Coquille *et al.*, 2014 ▸; Gully, Shah *et al.*, 2015 ▸). Crystallographic analysis has been particularly valuable for understanding the molecular basis of sequence-specific RNA recognition and the dramatic conformational changes that accompany RNA binding. This review provides a comprehensive catalogue of all PPR crystal structures deposited in the Protein Data Bank through 2024, analysing their crystallographic properties and contributions to our understanding of PPR function.

## 2. PPR protein architecture and classification

**Figure 1:**
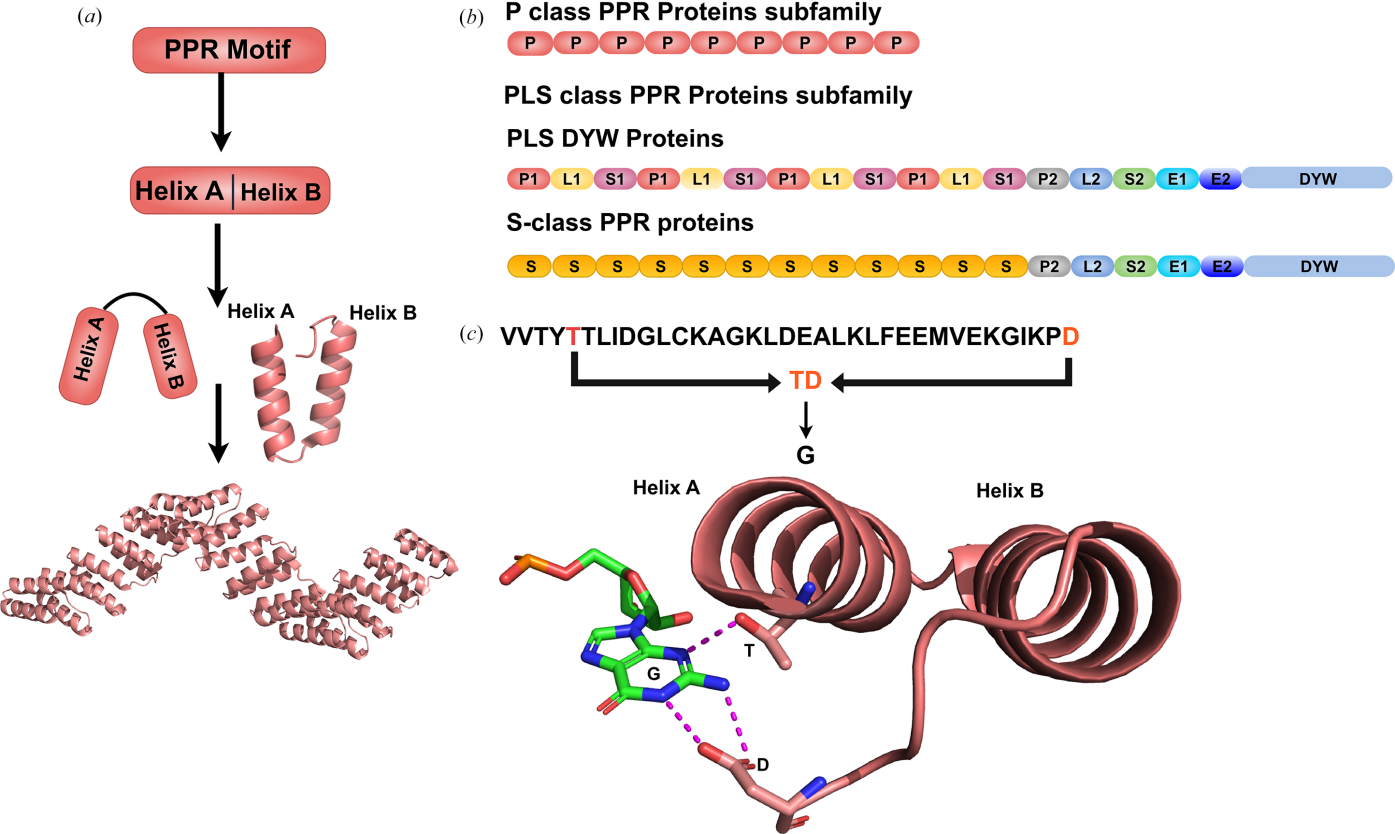
Key features of pentatricopeptide repeat proteins. (*a*) Schematic of the PPR motif showing two antiparallel α-helices (helix A and helix B), which are arranged in a helix–turn–helix arrangement, subsequently forming a superhelical structure. (*b*) Schematic representation of the domain architecture of the P-class and PLS-class subfamilies, including PLS DYW proteins and S-class PPR proteins. (*c*) Recognition of a single nucleotide base (G) with the fifth and 35th amino-acid residues (T and D). Hydrogen bonds are shown as dashed magenta lines.

PPR proteins are classified into two major subfamilies based on their repeat composition (Fig. 1 ▸; Cheng *et al.*, 2016 ▸). P-class proteins contain only canonical P-type repeats of 35 amino acids each. PLS-class proteins contain a mixture of canonical P repeats (35 residues), long L repeats (35–36 residues) and short S repeats (31 residues), often terminating in a DYW domain with cytidine deaminase activity (Cheng *et al.*, 2016 ▸; Lurin *et al.*, 2004 ▸; Li *et al.*, 2021 ▸; McDowell *et al.*, 2022 ▸; Fig. 1 ▸*b*). These proteins are composed of PLS repeats, which include repeats of P1, L1, S1 followed by P2, L2, S2 motifs. P1 and P2 are canonical P motifs with 35 amino acids that differ slightly in the first helix. L1 and L2 tend to be slightly longer (35–36 amino acids), with key differences in the second helix. S1 and S2 are smaller motifs of 31 and 32 amino acids, respectively (Cheng *et al.*, 2016 ▸; Wang *et al.*, 2021 ▸).

Each PPR repeat adopts a helix–turn–helix motif consisting of two antiparallel α-helices (designated helix A and helix B) connected by a short turn (Yin *et al.*, 2013 ▸; Fig. 1 ▸*a*). These repeats stack to form a right-handed superhelical solenoid with a concave RNA-binding surface (Yin *et al.*, 2013 ▸). The modular nature of this architecture enables sequence-specific RNA recognition through a ‘PPR code’ wherein amino acids at specific positions within each repeat determine the identity of the cognate RNA base (Barkan *et al.*, 2012 ▸; Fig. 1 ▸*c*).

## 3. Survey of PPR crystal structures

Since the first PPR protein crystal structures were published in 2013, there has been a steady flow of new structures both in the context of individual native proteins, individual ‘designer’ proteins and PPR proteins involved in large complexes such as ribosomes, which have been studied by a variety of techniques including atomic-resolution crystallography and cryo-electron microscopy (cryo-EM) as surveyed here, but complemented by computational studies, solution small-angle X-ray scattering (SAXS), single-molecule Förster resonance energy transfer and other related methods. In this survey, we describe experimental structures incorporating at least one PPR motif according to the InterPro PPR definition (IPR002885; Blum *et al.*, 2025 ▸) and supplement this information with additional structures identified as PPR proteins by the authors of relevant articles. A summary of selected representative PPR crystal structures is given in Table 1 ▸.

### 3.1. Native PPR protein structures

#### 3.1.1. *Zea mays* PPR10: the first experimental PPR structures

*PDB entries 4m57, *Zea mays* PPR10 (apo form), 4m59, *Z. mays* PPR10 in complex with 18 nt *psaJ* RNA, and 4oe1, *Z. mays* PPR10 (cysteine variant) with *psaJ* RNA.*

**Figure 2:**
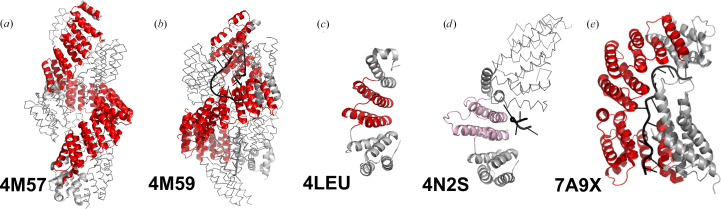
Structures of native plant and yeast PPR proteins. (*a*, *b*) *Zea mays* PPR10 in the absence and presence of RNA. (*c*) THA8-like protein from *Arabidopsis thaliana*. (*d*) THA8 protein from *Brachypodium distachyon* in complex with RNA. (*e*) Yeast Rmd9p protein in complex with RNA. All molecular graphics were prepared with *PyMOL* (Schrödinger). One protomer is displayed as a cartoon. InterPro-defined PPR motifs are coloured red and manually curated PPR-like motifs are shown in pink. Nucleic acid is shown as a black cartoon. Additional protomers (crystallographically or non­crystallographically related, where appropriate) are shown as grey ribbons.

The structure of maize PPR10 represents an important point in PPR structural biology, providing the first experimental structure of a PPR protein (PDB entry 4m57; Yin *et al.*, 2013 ▸; Fig. 2 ▸*a*). The structure revealed 19 ‘P-class’ PPR repeats arranged in a right-handed superhelix with dimensions of 140 × 70 Å. The structure established key architectural principles: each PPR motif contains ∼35 amino acids forming two antiparallel α-helices, with helix A comprising residues 1–16 and helix B comprising residues 17–35. The superhelical parameters (rise per repeat, 7.5 Å; twist, 19°) create a positively charged concave surface ideal for RNA binding (Yin *et al.*, 2013 ▸).

Yin and coworkers revealed the structure of PPR10 in complex with single-stranded RNA (PDB entry 4m59; Fig. 2 ▸*b*), providing structural insight into the PPR code (Yin *et al.*, 2013 ▸) and PPR–RNA recognition in general. Binding to an oligonucleotide derived from *psaJ* mRNA from maize (one of its two *in vivo* targets) induces a dramatic 20 Å compression of the superhelix, demonstrating the dynamic structural repertoire of PPR proteins (Yin *et al.*, 2013 ▸). Six nucleotides show sequence-specific recognition following the predicted PPR code, with the fifth amino acid of each repeat making specific contacts with the Watson–Crick edge of the cognate base (Yin *et al.*, 2013 ▸).

The PPR10 and PPR10–RNA complexes crystallized as a protein homodimer, although solution studies (Barkan *et al.*, 2012 ▸; Gully, Cowieson *et al.*, 2015 ▸) suggest this may not be fully reflective of the behaviour of PPR10 in solution (see Section 4.2). In order to obtain crystals, PPR10 had undergone a wholesale mutagenesis of cysteine to serine, with one construct (C256S/C279S/C430S/C449S) giving rise to crystals. PDB entry 4oe1 (Li *et al.*, 2014 ▸) recapitulates the structure of PDB entry 4m59 with position 279 unmutated, as part of an investigation of the role of mutation to serine in biasing the oligomeric state.

#### 3.1.2. THA8 family proteins

*PDB entries 4leu, *Arabidopsis thaliana* THA8-like protein, 4me2, *Brachypodium distachyon* THA8 (apo form), 4n2q, THA8 in complex with Zm4 RNA (GGGG), and 4n2s, THA8 in complex with Zm1a-6 RNA (GGGAG).*

PDB entry 4leu represents the first crystallographic characterization of a PPR protein with a more diverse set of repeats, solved using SeMet single-wavelength anomalous diffraction (Fig. 2 ▸*c*; Ban *et al.*, 2013 ▸). While the nominal ‘LPPPS’ repeat architecture is not a ‘PLS-type’ PPR protein in a biological sense, this structure demonstrated key differences from typical P-class PPR proteins. The so-called L motif is composed of an extended helix A and a shortened helix B, which differs from the structures of true L motifs found in later crystal structures. The asymmetric positive-charge distribution on the protein surface suggested a highly specific RNA-binding orientation (Ban *et al.*, 2013 ▸).

A subsequent study determining structures of the THA8 protein from *B. distachyon*, both without and with short maize RNA sequences derived from a cross-species binding assay, revealed that AGAAA RNA induces an asymmetric dimerization of THA8 for high-affinity binding involving many noncanonical interactions (Fig. 2 ▸*d*). This work indicated that not all naturally occurring PPR proteins may bind RNA using the canonical ‘PPR code’ (Ke *et al.*, 2013 ▸).

#### 3.1.3. Yeast Rmd9 structures

*PDB entries 7a9x, *Saccharomyces cerevisiae* Rmd9p with 16 nt RNA, and 7a9w, *S. cerevisiae* Rmd9p with 20 nt RNA.*

The structures of the yeast mitochondrial PPR protein Rmd9p in complex with RNA (Fig. 2 ▸*e*) further demonstrated that PPR proteins have adapted to modified RNA-recognition modes (Hillen *et al.*, 2021 ▸). The architecture of Rmd9p includes a superficially PPR-like N-terminal ‘lid’ domain which folds back over the central tract of eight PPR motifs to create a series of alternate contact points with the RNA, thus only following the canonical binding mode for two of the motifs. The C-terminal domain is a non-PPR helical bundle. The RNA follows a path through the protein interior rather than along the concave surface, with extensive base-stacking interactions rather than sequence-specific hydrogen bonding. This represents a fundamentally different recognition strategy from the canonical PPR code, and illustrates some of the challenges that may be encountered in predicting RNA-binding sites from protein structure (Hillen *et al.*, 2021 ▸).

#### 3.1.4. Proteinaceous RNase P structures

*PDB entries 4g23, 4g24, 4g25 and 4g26, *Arabidopsis thaliana* proteinaceous RNase P 1 (*At*ProRP1) with Mn^2+^,* *Ca^2+^ and Sr^2+^ ions, 6bv5, 6bv6, 6bv8 and 6bv9, *At*ProRP1 complexed with juglone, and 6lvr, *At*ProRP1 complexed with tRNA.*

Proteinaceous RNase P (ProRP) enzymes represent an evolutionary innovation where protein-only enzymes have replaced the ancient ribonucleoprotein RNase P complexes for 5′-end processing of precursor tRNAs, particularly in the nucleus and mitochondria of plants and animals (Gutmann *et al.*, 2012 ▸). The nine crystal structures deposited since 2012 have revealed a unique tridomain architecture combining a domain containing a single pentatricopeptide repeat (PPR) for RNA binding, a structural zinc-binding module and an NYN metallonuclease domain that catalyses phosphodiester-bond hydrolysis through a two-metal-ion mechanism. Key structural insights include the first atomic structures, incorporating Mn^2+^, Ca^2+^ or Sr^2+^ ions (PDB entries 4g23, 4g24, 4g25 and 4g26; Howard *et al.*, 2012 ▸), and the mechanism of covalent inhibition through time-resolved juglone-modification studies (PDB entries 6bv5, 6bv7, 6bv8 and 6bv9). The substrate-recognition mode revealed by the ProRP1–tRNA complex (PDB entry 6lvr) shows how these enzymes achieve broad substrate specificity unlike typical sequence-specific PPR proteins.

#### 3.1.5. Mitochondrial subunits

*PDB entries 3j9m, 6neq, 6nf8, 6nu2, 6nu3, 6rw4, 6rw5, 6vlz, 6vmi, 6zm5, 6zm6, 6zs9, 6zsa, 6zsb, 6zsc, 6zsd, 6zse, 6zsg, 7a5f, 7a5g, 7a5i, 7a5k, 7l08, 7og4, 7p2e, 7pnx, 7pny, 7pnz, 7po0, 7po1, 7po2, 7po3, 7qi4, 7qi5, 7qi6, 8any, 8csp, 8csq, 8csr, 8css, 8cst, 8csu, 8k2a, 8oir, 8ois, 8qrk, 8qrl, 8qrm, 8qrn, 8rri, 8xt0 and 8xt2, human mitochondrial ribosome, 6gaw, 6gaz, 6ydp, 6ydw, 7nqh, 7nql, 7nsi, 7nsj, 8oin and 8oip, *Sus scrofa*/human mitochondrial ribosome, 3jd5, *Bos taurus* mitochondrial ribosome, 7pnt, 7pnu, 7pnv and 7pnw, *Mus musculus* mitochondrial ribosome, 6xyw, *Arabidopsis thaliana* mitochondrial ribosome, 6hiv, 6hix, 6sga, 6sgb, 6yxx, 6yxy, 7aoi, 7pua, 7pub and 9hny, *Trypanosoma brucei* mitochondrial ribosome, 7am2 and 7ane, *Leishmania major* mitochondrial ribosome, 7aih, *L. major* kinetoplastid ribosome, and 8d8j and 8d8k, *Saccharo­myces cerevisiae* mitochondrial ribosome assembly intermediates.*

These PPR-protein-containing PDB entries are all eukaryotic mitochondrial ribosomes, including complete ribosomes, subunits and assembly intermediates.

**Figure 3:**
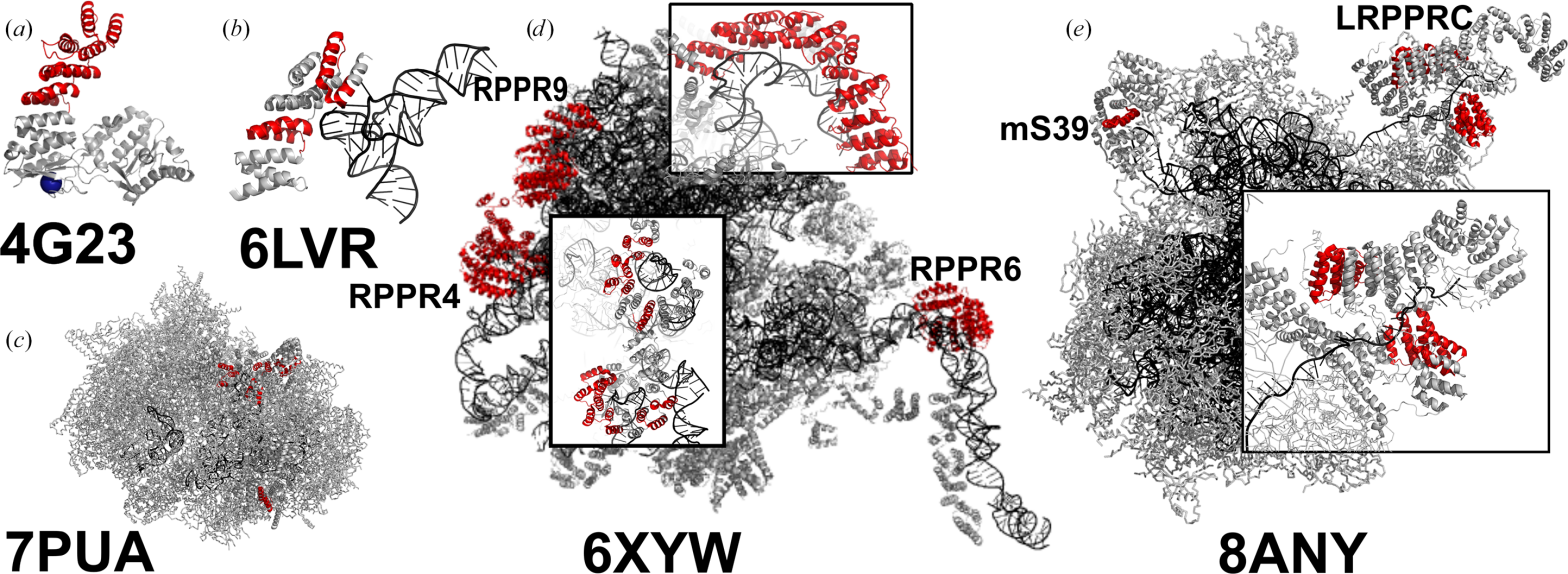
Structures of ProRP and mitochondrial ribosomes. (*a*, *b*) *At*ProRP1 in the absence and presence of tRNA. (*c*) Assembly intermediate of *T. brucei* mitochondrial ribosome. (*d*) *A. thaliana* ribosome. All PPR proteins are shown in red. Insets show the principal PPR subunits RPPR4 and RPPR9, and RPPR6. (*e*) Human mitochondrial ribosome. Inset: PPR-containing subunit mS39 and LRPPRC.

In number, mitochondrial ribosome PPR components and accessories represent the largest structural dataset in PPR protein research, but with substantial redundancy. These structures are predominantly from human mitochondrial ribosomes (52) but also include examples from cow, pig, mouse, yeast, plants and parasitic protozoa. In most cases, PPR motifs are found in isolation (exemplified in Fig. 3 ▸*c*), but a small number of PPR proteins with tracts of repeats are also observed. The *A. thaliana* mitochondrial ribosome includes six ribosomal PPR (RPPR) proteins (RPPR4, RPPR6 and RPPR9 are identified by InterPro and highlighted in Fig. 3 ▸*d*). The human mitochondrial ribosome includes the PPR-tract-containing mS39 subunit and interacts with LRPPRC (Fig. 3 ▸*e*). In general, ribosomal PPR proteins are involved in inter­actions with both single-stranded and structured RNA but often in noncanonical ways, so that these interactions tend not be particularly informative for predicting or understanding PPR–RNA interactions in other contexts.

### 3.2. Consensus or designer PPR proteins (cPPR and dPPR)

#### 3.2.1. Designer apo P-class PPR structures

*PDB entries 4ozs, 3.5 motif consensus poly-U RNA-binding PPR protein (Gully, Shah *et al.*, 2015 ▸), 4pjq, 4wn4, 4wsl, 4pjr and 4pjs (Coquille *et al.*, 2014 ▸), 8.5 motif consensus PPR proteins targeting poly-G, poly-A, poly-C or nanos response element (NRE) RNA, and 5orm, 10.5 motif consensus PPR protein targeting telomeric ssDNA (Spåhr *et al.*, 2018 ▸).*

**Figure 4:**
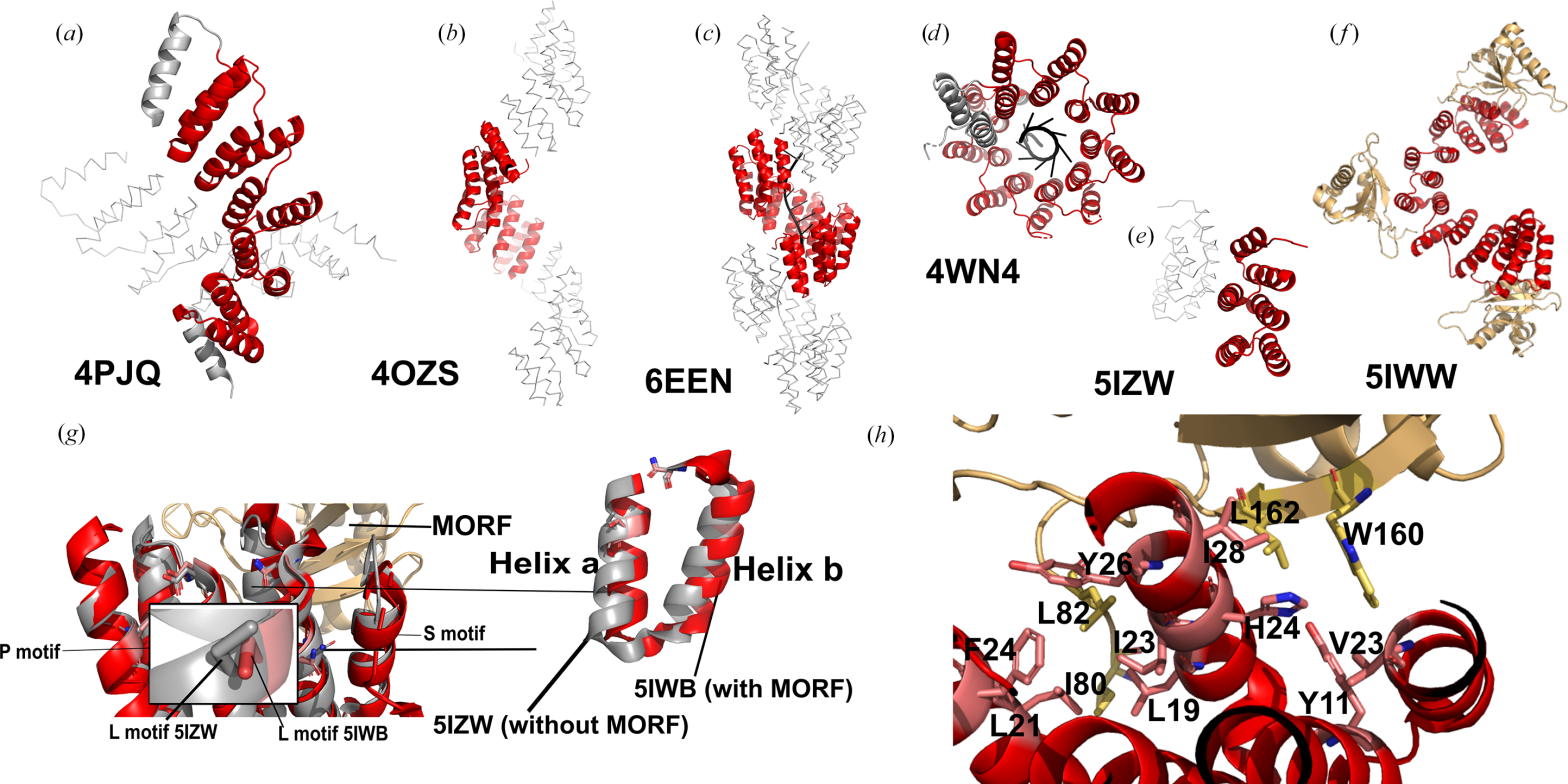
Structures of designer PPR proteins and their complexes. (*a*, *b*) Consensus P-class repeat proteins in the absence of RNA. (*c*, *d*) Consensus P-class repeat proteins in the presence of RNA. (*e*, *f*) Consensus PLS-class proteins in the absence and presence of MORF cofactor. MORF is labelled in light orange. (*g*) Superimposed structure of PLS-class proteins in the presence and absence of MORF cofactor, with P, L and S motifs labelled. Orientation of the fifth amino acid in PDB entry 5izw (grey) without MORF and PDB entry 5iwb (red) with MORF are zoomed in. Helices a and b of L motifs are zoomed in to show the difference in their orientation of the motifs upon binding MORF protein. (*h*) Residues involved in the interactions between MORF and P, L and S motifs (adapted from Yan *et al.*, 2017 ▸).

This group of structures comprise the first ‘designer’ PPR proteins, whose sequences are based on monotonous repeats of the consensus sequence of naturally occurring P-class motifs, in the manner previously pioneered for tetratricopeptide repeat proteins (Main *et al.*, 2003 ▸). A modified half-repeat is appended to the C-terminus as a solubilizing helix. Using the PPR code (Barkan *et al.*, 2012 ▸), amino acids at the fifth and 35th positions are altered to specify recognition of a desired base by that motif. PDB entries 4ozs, 4pjq (Fig. 4 ▸*a*), 4wn4 (Fig. 4 ▸*d*) and 4wsl all target homopolymers, while PDB entries 4pjr, 4pjs and 5orm target complex native sequences. PDB entries 4ozs (Fig. 4 ▸*b*), 4pjr (Fig. 4 ▸*a*) and 4pjs are isomorphous and extremely similar in sequence. PDB entry 5orm is unique in the set in that it was targeted to ssDNA rather than ssRNA (discussed in Section 3.2.2).

These studies established that designer PPR proteins are more soluble and stable in solution than native PPR proteins, and that their structures confirm the expected right-handed superhelical arrangement predicted previously (Fujii & Small, 2011 ▸). A specific type of helical disorder (Supplementary Fig. S1) observed in all of these structures (excepting PDB entry 4wsl) results in the solubilizing helix not being observed in the structure, alongside other complications which are discussed in Section 4.3.

#### 3.2.2. Designer P-class PPRs in complex with nucleic acids

*PDB entries 5i9d, 5i9f, 5i9g and 5i9h, 10.5 motif consensus P-class PPR proteins in complex with U8A2, U10, U8C2 or U8G2 RNA, respectively (Shen *et al.*, 2016 ▸), 5orq, 10.5 motif consensus P-class PPR protein in complex with a telomeric ssDNA sequence (Spåhr *et al.*, 2018 ▸), and 6een, 17.5 motif consensus P-class PPR protein in complex with a 19-base oligonucleotide from *A. thaliana* tph RNA (Fig. 4 ▸*c*; Marzano *et al.*, 2024 ▸)*.

This group of structures of PPR protein–nucleic acid complexes confirmed the PPR code mode of interaction of residues 5 and 35 of consecutive motifs with consecutive bases in an oligonucleotide, arranged in a 5′–3′ direction with respect to the protein sequence (Shen *et al.*, 2016 ▸). PDB entries 5i9d, 5i9f, 5i9g and 5i9h targeted RNA comprising *xx*UUUUNNUUUU*xx*, where N is U, A, C or G and *x* indicates some additional nucleotides. PDB entry 5orq uses the same telomeric ssDNA-binding protein as PDB entry 5orm above, in complex with ssDNA, revealing that the same rules apply for ssDNA binding as ssRNA, albeit with impaired affinity. The protein used for PDB entry 6een mimics the binding of *At*PPR10 (Fig. 4 ▸*c*), but with a designer PPR protein. The long 17 nt recognition sequence results in high affinity; however, the structure also displays helical disorder which obscures the detail of amino acid–base interactions (discussed in Section 4.3).

A major observation from this group of structures is that binding of the spring-like superhelical PPR array results in compression of the spring by a factor of close to two. This is discussed in Section 4.2.

#### 3.2.3. Designer PLS-class PPR proteins

*PDB entries 5iwb, consensus PLS triplet PPR protein complexed with MORF (multiple organellar RNA-editing factor) protein (Fig. 4 ▸*e*), 5iww, consensus (PLS)_3_ protein* *complexed with three MORF proteins (Fig. 4 ▸*f*), and 5izw, consensus PLS triplet in the absence of MORF protein.*

This group of structures, described by Yan and coworkers, are currently the only examples of structural data on a bona fide PLS-class PPR protein. Using a similar kind of consensus protein chassis as for P-class proteins, structures were obtained in the absence and presence of MORF adaptor proteins, which have been demonstrated to be important for RNA binding by some native PLS proteins. They reveal the mechanism by which MORF proteins modulate PLS–PPR function (Yan *et al.*, 2017 ▸). MORF proteins bind to the L motifs, stabilizing the orientation of the motif through hydrophobic packing (Fig. 4 ▸*h*) and electrostatic interactions, which result in reorganization of the helix–turn–helix motifs. In the apo PLS–PPR structure, L motifs adopt an ‘outward’ orientation with helix B rotated away from the RNA-binding surface, creating excessive distance between the fifth and 35th positions for effective base contact (the rotation of the fifth amino-acid residue is depicted in Fig. 4 ▸*g*). MORF9 binding induces a dramatic conformational change, rotating L-motif helix B inwards by ∼6° and bringing the recognition residues into proper geometry for RNA binding.

### 3.3. Structures of DYW domains

*PDB entries 7o4e and 7o4f, DYW domain of *A. thaliana* OTP86 (‘inactive’ and ‘active’ forms; Takenaka *et al.*, 2021 ▸), and 7w86, DYW domain of *A. thaliana* DYW1 (Toma-Fukai *et al.*, 2023 ▸).*

The DYW domain is an RNA-editing enzyme domain that is uniquely associated with PPR proteins, although the domain itself does not include PPR repeats (PDB entries 7o4e and 7o4f).

**Figure 5:**
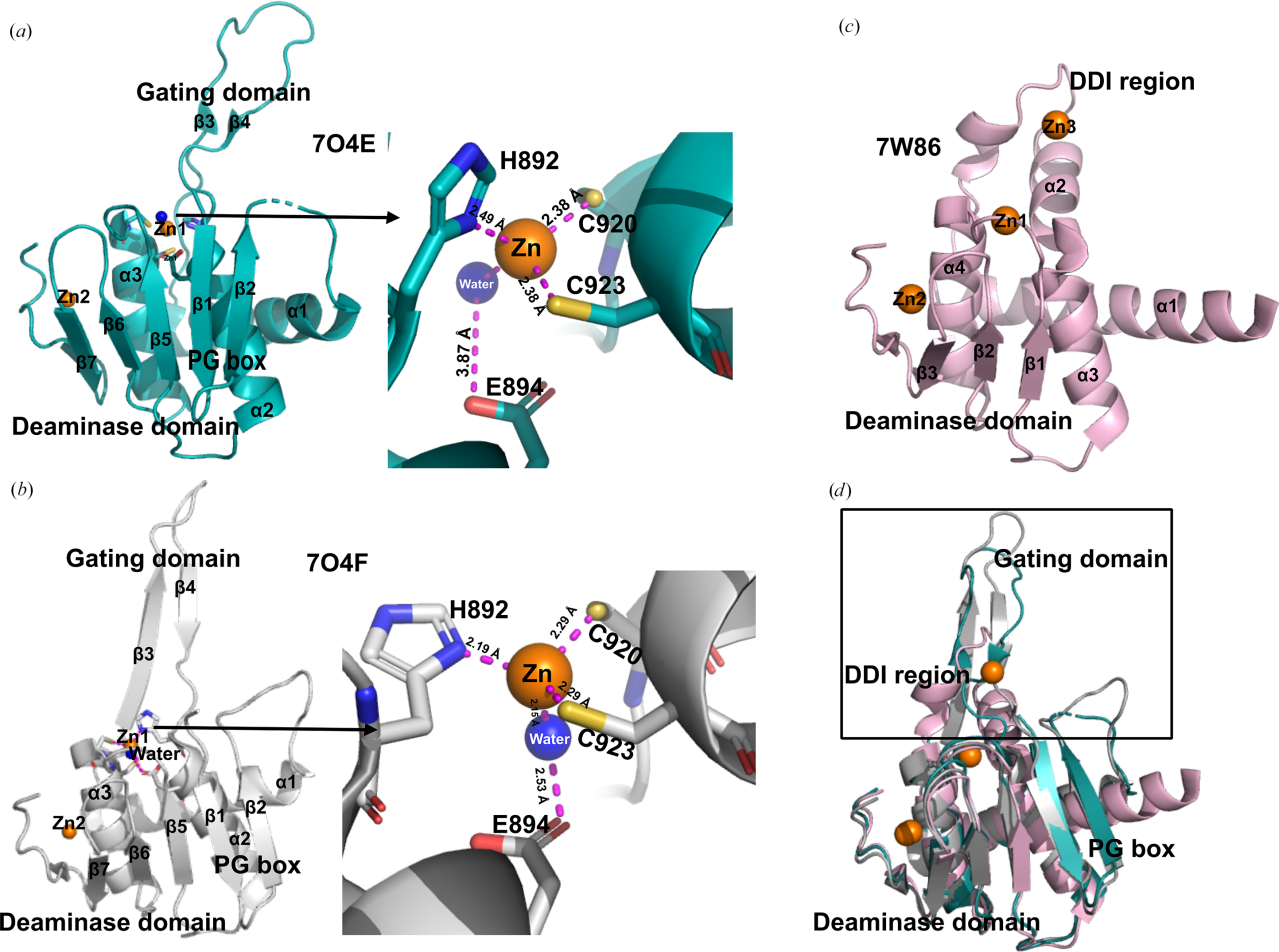
(*a*) PDB entry 7o4e in pale cyan showing the PG box, gating domain, deaminase domain and DYW motif. (*b*) PDB entry 7o4f: active conformation of DYW with the extended gating domain shown in grey. (*c*) PDB entry 7w86 in pale pink showing the deaminase domain and DDI region. (*d*) Superimposed models of crystal structures of DYW domains [inactive (PDB entry 7o4e) in deep teal, active (PDB entry 7o4f) in grey and DYW1 (PDB entry 7w86) in pale pink].

Solved using single-wavelength anomalous dispersion, the *At*OTP86 DYW domain was the first experimental evidence that the DYW domain structure is structurally related to the canonical cytidine deaminase domain, albeit with additional characteristics. The authors reported conformational differences between the two structures, PDB entries 7o4e and 7o4f, which they identify as inactive and active conformations, as a mechanistic explanation for how RNA-editing activity could be allosterically regulated to limit off-target editing. OTP86 has a cytidine deaminase-like sequence signature, H*x*EC*xx*C, and both structures have five β-sheets flanked by two α-helices which superimpose well (r.m.s.d. of 1.89 Å) with other cytidine deaminase proteins. This domain contains two zinc ions; one is located in the active site, while the second zinc ion is unique to DYW proteins and appears to be purely structural. The active-site zinc ion is assumed to activate water in the deamination reaction through the involvement of His892, Cys920, Cys923 and Glu894. Novel features of the DYW domain include a so-called ‘PG box’ sequence motif, which may relate to how the domain interacts with the PPR tract of the rest of the protein, and a ‘gating domain’. This region is extended in both structures, as a loop in PDB entry 7o4e and as a β-hairpin in PDB entry 7o4f (Figs. 5 ▸*a*, 5 ▸*b* and 5 ▸*c*). These conformational changes may reflect activation of the enzyme activity as a result of movement in the protein backbone which causes a reorganization of the DYW active site (Takenaka *et al.*, 2021 ▸). However, in the ‘active’ crystal structure PDB entry 7o4f, the gating domain β-hairpin forms a β-sheet with a crystallo­graphic neighbour molecule, which is an unlikely event in the native environment. The structural differences in this region between the two crystal structures casts some uncertainty on how it might regulate enzyme activity.

In addition to its varied secondary structure, PDB entry 7w86 (DYW1; also solved by SAD) adds to the structural gating story, as the DYW1 protein is a truncated DYW domain that requires complementation by CRR4 to form a heterodimer protein that can act like other PPR–DYW holoproteins (Boussardon *et al.*, 2012 ▸). In DYW1 the deaminase domain is composed of four β-sheets and two α-helices, and it lacks the PG box. The gating domain (referred to as the DDI region in this study) adds further evidence of structural divergence, being composed of α-helix 1, a 3_10_-helix and α-helix 2 (Fig. 5 ▸*d*). The coordination of the zinc ions is similar to that of OTP86. However, this structure includes a third zinc ion which is likely to be an artefact of crystallization (Toma-Fukai *et al.*, 2023 ▸).

## 4. Structural analysis and insights

### 4.1. Canonical and noncanonical interactions in PPR proteins

PPR proteins can contact RNA via canonical or non­canonical interactions. We define a canonical interaction with an RNA base as one that can be described by the PPR code (Barkan *et al.*, 2012 ▸) and a noncanonical interaction as one that cannot.

#### 4.1.1. The PPR recognition code: structural validation

Crystallographic studies of both native and designer PPR proteins have provided both definitive validation of the PPR recognition code initially proposed from bioinformatics analyses (Barkan *et al.*, 2012 ▸), as well as examples of diversity in RNA binding that does not feature in the PPR code. In the studies that established the code, different numbering schemes for motifs were used to identify the key specifying residues, including positions 5 and 35 (Yin *et al.*, 2013 ▸; Cheng *et al.*, 2016 ▸), residues 6 and 1′ (Barkan *et al.*, 2012 ▸) and 4 and ii (Yagi *et al.*, 2013 ▸), but the field has converged on ‘fifth and last’ to also provide a valid definition for PPR motifs of lengths other than the P-class 35, such as L and S motifs. Side chains at these two positions form hydrogen bonds to the Watson–Crick edge of the target base either directly or through an interstitial water molecule. In some cases, to assist binding of purines, a larger base at position 2 can intercalate between bases and thus modify the code (Yin *et al.*, 2013 ▸). The code shows remarkable consistency across all PPR proteins, but many native proteins, including the structural exemplars PPR10, Rmd9 and THA8, have also evolved noncanonical inter­actions that increase the selectivity and affinity for their target.

Ultimately, the direct application of canonical formalisms such as the PPR code to native PPR proteins may be limited due to the additional quirks they have evolved; however, the code is being revealed to be extremely effective in the creation of designer proteins aimed at an RNA target of choice (Shen *et al.*, 2016 ▸; Bernath-Levin *et al.*, 2021 ▸; Dennis *et al.*, 2025 ▸; Marzano *et al.*, 2024 ▸; Coquille *et al.*, 2014 ▸).

#### 4.1.2. Noncanonical binding in PPR proteins

**Figure 6:**
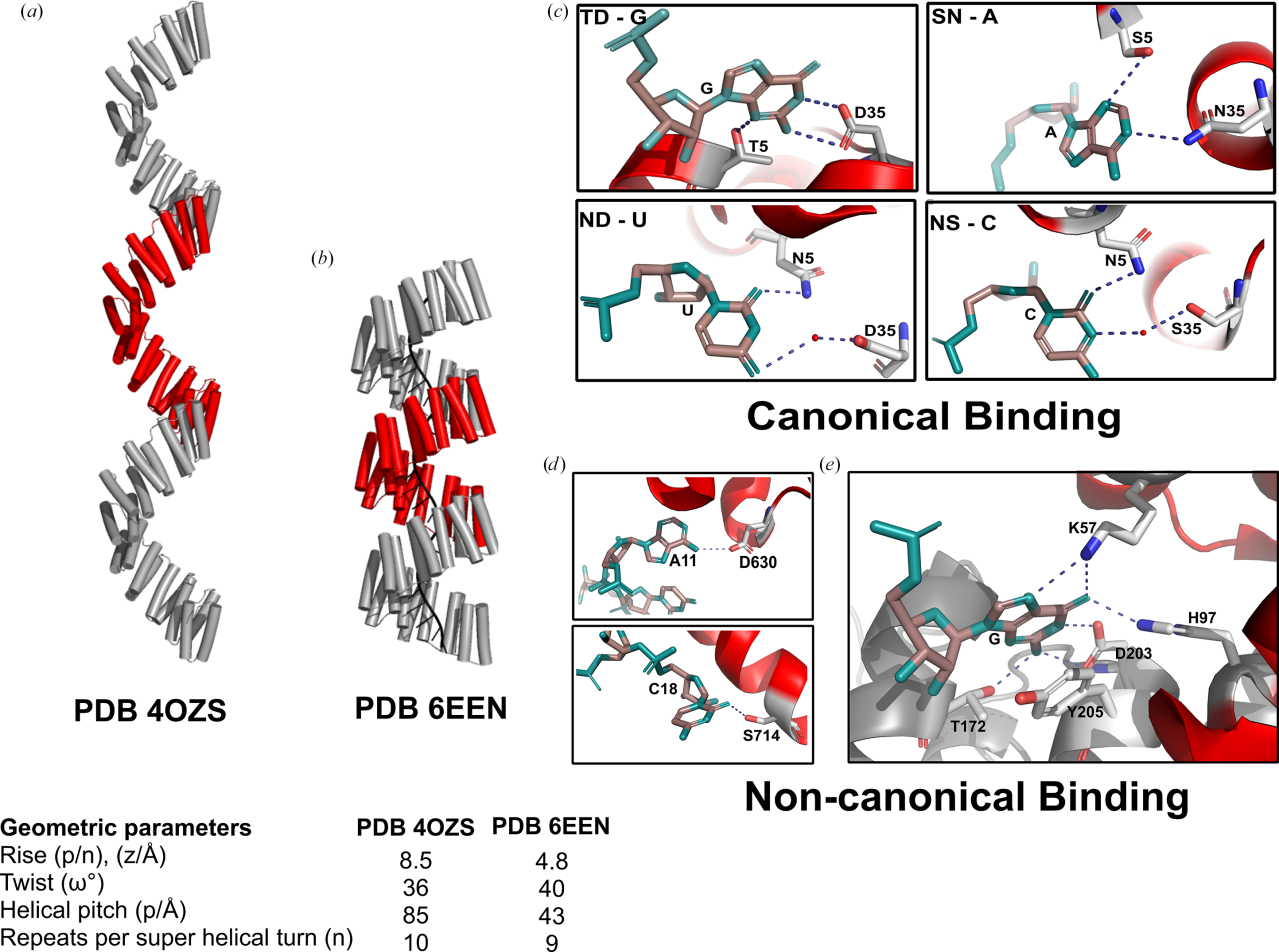
(*a*) Superhelical structure of apo designer P-class PPR protein (PDB entry 4ozs); one helical repeat is shown in red (adapted from Marzano *et al.*, 2024 ▸). (*b*) Superhelical structure of designer P-class PPR protein bound to RNA (PDB entry 6een). (*c*) Canonical binding of PPR proteins with the nucleotide bases guanine (G), adenine (A), uracil (U) and cytidine (C). The fifth and 35th amino-acid residues in each repeat are highlighted as sticks. The hydrogen-bonding interaction is shown in blue. Water molecules are depicted as red spheres (adapted from Shen *et al.*, 2016 ▸). (*d*) Noncanonical binding of PPR10 protein, showing hydrogen bonding between adenine (A) and aspartate (D) 630 and between cytidine and serine (S) 714 (adapted from Yin *et al.*, 2013 ▸). (*e*) Interaction of THA8 dimer proteins with Zm4 RNA nucleotide base G. Hydrogen bonds are shown as blue dashed lines. The amino acids and nucleotide bases are shown as sticks (adapted from Ke *et al.*, 2013 ▸).

Noncanonical binding of PPR proteins refers to interactions that deviate from the PPR code and which generally arise from atypical side-chain contacts. For example, in the PPR10–*psaJ* (PDB entry 4m59) interaction, Asp630 (the 35th residue of the 15th repeat) hydrogen bonds to A11’s exocyclic N6 amino atom, resulting in specificity towards adenine instead of uridine as predicted by the PPR code (Fig. 6 ▸*d*; Yin *et al.*, 2013 ▸). In yeast, Rmd9 (PDB entry 7a9x) forms a tunnel with an N-terminal lid, eight PPR motifs and C-terminal domains to interact with conserved 12 nt dodecamer RNA via base-stacking interactions (Hillen *et al.*, 2021 ▸). THA8 forms a dimeric interface, enabling RNA binding through base stacking and backbone interactions with Arg/Lys residues rather via canonical interactions (Fig. 6 ▸*e*; Ke *et al.*, 2013 ▸). These examples indicate that the inter-motif organization can modulate RNA recognition beyond sequence-specific modular binding.

### 4.2. Superhelical architecture and flexibility

Structural studies have established that PPR proteins adopt a characteristic right-handed superhelical architecture with remarkable consistency across both native and designer proteins. A fascinating observation was that the superhelical parameters vary substantially depending on RNA-binding state. A direct comparison between designer PPR proteins composed of almost identical repeats, with only positions 5 and last varying, either alone (PDB entry 4ozs; Gully, Shah *et al.*, 2015 ▸) or in complex with RNA (dPPR10; PDB entry 6een; Marzano *et al.*, 2024 ▸), reveals a remarkable overwinding and compression of the superhelix, with the number of repeats per superhelical turn reducing from ten to nine (helical twist changing from ∼36° to ∼40°) and the helical pitch decreaing from ∼85 to ∼43 Å (rise changing from ∼8.5 to ∼4.8 Å). This compression had been observed in SAXS and analytical ultracentrifugation studies of *At*PPR10 and RNA in a study (Gully, Cowieson *et al.*, 2015 ▸) complementary to the *At*PPR10 structures PDB entries 4m57 and 4m59 (Yin *et al.*, 2013 ▸). Furthermore, these observations inspired a single-molecule Förster resonance energy transfer (FRET) experiment where complementary maleimide-functionalized Cy3 and Alexa Fluor 647 dyes were attached to cysteine residues that were introduced to dPPR10 using PDB entries 4ozs and 6een as a guide (Marzano *et al.*, 2024 ▸). Clear changes in FRET were used to analyse the binding behaviour of populations of hundreds of individual immobilized dPPR10 molecules. This dramatic compression upon RNA binding (a nearly 50% reduction in pitch) represents one of the largest conformational changes observed in any RNA-binding protein family (Marzano *et al.*, 2024 ▸). The compression may be facilitated by conserved lysine residues at position 13 that form salt bridges with RNA phosphates and couple RNA binding to structural reorganization (Coquille *et al.*, 2014 ▸). However, we note that S-class motifs do not contain a lysine at this position, suggesting that either the lysine residue is not critical for the compaction mechanism or that S-class proteins may operate differently.

#### 4.2.1. Mechanism of MORF-mediated activation

The limited available structural data on PLS-class protein partners reveal an elegant allosteric mechanism for controlling RNA-binding affinity. The structure of the PLS protein alone is not optimal for RNA binding, but conformational change induced by binding of a MORF protein to the L motif leads to the inward orientation of L motif by approximately 6°, bringing the 35th amino-acid residue close to the fifth amino acid, permitting sequence-specific binding.

The inherent flexibility of PPR proteins revealed by these studies suggests that structure predictions may have reduced applicability in some circumstances, as the prediction may lie anywhere on a continuum from compressed to extended. However, the fact that single-molecule studies are now possible opens the door to studies of the precise mechanisms of RNA binding.

### 4.3. Crystallographic considerations and solution behaviour

Several PPR structures exhibit crystallographic features and artefacts that initially complicated interpretation.

#### 4.3.1. Dimerization artefacts

While the pioneering PPR10 crystal structures showed protein homodimers in the absence and presence of RNA, including an unusual *trans*-binding effect where RNA was coordinated by both monomers, solution studies have clearly shown that PPR10 forms a compact monomeric (in terms of protein composition) complex on binding RNA (Gully, Cowieson *et al.*, 2015 ▸), suggesting that the observed dimer may have been a consequence of the construct used for crystallization (Li *et al.*, 2014 ▸). The oligomerization state for the unbound protein is less certain, with solution studies suggesting that more general aggregation rather than specific dimerization may be occurring (Gully, Cowieson *et al.*, 2015 ▸).

#### 4.3.2. Crystal packing and helical disorder

By creating proteins with identical PPR repeats, one creates the possibility that two PPR proteins can come together using the same repeated interface that occurs between repeats in the same protein. Such oligomers have been shown to form in solution from concentrated protein samples, which even resist partial denaturation on sodium dodecyl sulfate polyacrylamide gel electrophoresis (Gully, Shah *et al.*, 2015 ▸). This feature is observed in almost all designer PPR protein apo structures determined thus far and also in the dPPR10–*atpH* structure PDB entry 6een. Each of these examples shows a molecule spanning the asymmetric unit where it meets a new molecule and the superhelical arrangement continues. In these cases, the C-terminal solubilizing helix is absent from the electron density, presumably having been displaced by the intermolecular interaction. In most of the examples it is even more complicated, as the number of PPR repeats in the asymmetric unit does not match the number of repeats in the protein that was crystallized. For example, in the study reporting PDB entry 4ozs, proteins composed of 3.5 or 5.5 repeats produced isomorphous crystals, with structures that show a kind of helical disorder that results in electron density resembling an infinite protein superhelix. In this case, the density is excellent for determining atomic positions of this notional infinite protein and measuring superhelical parameters. In the case of dPPR10–*atpH* (PDB entry 6een), the same helical disorder is observed (a 17.5 repeat protein but with nine repeats spanning the asymmetric unit), which results in excellent electron density for the protein and RNA backbones, but scrambling of the electron density at points of variation: *i.e.* each of the bases and positions 5 and last.

In summary, the conformational variation on binding RNA, the variable conformations of native PPR proteins, allosteric conformational regulation of RNA binding and the constraints of the crystal lattice conspire to suggest that cryo-electron microscopy studies might be the best approach to fully exploring the conformational dynamic properties of PPR proteins at high resolution.

### 4.4. DYW domain catalytic mechanism

The DYW domain structures of OTP86 and DYW1 (Fig. 5 ▸) have revealed a sophisticated catalytic mechanism, based on the archetypal cytidine deaminase enzyme chassis with a catalytic zinc ion, but with an additional structural zinc ion and a proposed structurally regulated gating mechanism to avoid off-target effects.

It is currently uncertain how the DYW domain interacts with the PPR region of the protein, and whether the structure of the gating domain is related to this interaction. Structural diversity in the gating domains suggests that the mechanism may not be completely uniform between PPR–DYW editing factors. It is also not known how the RNA substrate enters and leaves the active site, and which protein domains it interacts with as it passes through the protein. Therefore, we hypothesize that the large conformational change in the PPR tract upon binding RNA may alter the conformation of the gating domain, allowing editing to proceed.

### 4.5. *AlphaFold* predictions of PPR proteins

Nearly 75 *AlphaFold* predictions of PPR proteins from various species were retrieved from the *AlphaFold* database (listed in Supplementary Table S2; Varadi *et al.*, 2022 ▸, 2024 ▸) and crystal structures of PPR10 (PDB entries 4m59 and 5iwb) predicted using *AlphaFold*3 (Abramson *et al.*, 2024 ▸) were analysed to understand the structural differences between the experimental and predicted structures of PPR proteins. AlphaFoldDB provides a commonly used platform for biologists to access structural models, and so the quality and validity of these models is an important consideration. Where predictions of protein–RNA structures are required, *AlphaFold*3 is one of the most used tools (Abramson *et al.*, 2024 ▸). Across all these models, α-helical architecture was predicted with high confidence, with most residues showing a pLDDT score above 70 and some exceeding 85. The superhelical solenoid arrangement is well defined throughout the models, indicating that *AlphaFold* captures the repetitive motifs and interconnected linkers between helices A and B with very high confidence. In contrast, N-terminal organelle-targeting peptides, C-terminal extensions and intrinsically disordered and flexible regions were predicted to have low pLDDT scores and elevated predicted aligned error (PAE), indicating that *AlphaFold* is unsure about the structural arrangement of these residues. Another key observation was that the intra-domain PAE values for these proteins were consistently low (<5 Å), suggesting that *AlphaFold* has high confidence in the relative positioning of the individual motifs. These findings complement more detailed studies of AI-predicted PPR proteins which show consistency in the helical arrangement of the repeats, and interconnected linker regions that collectively form a superhelical structure (Barik, 2025 ▸).

**Figure 7:**
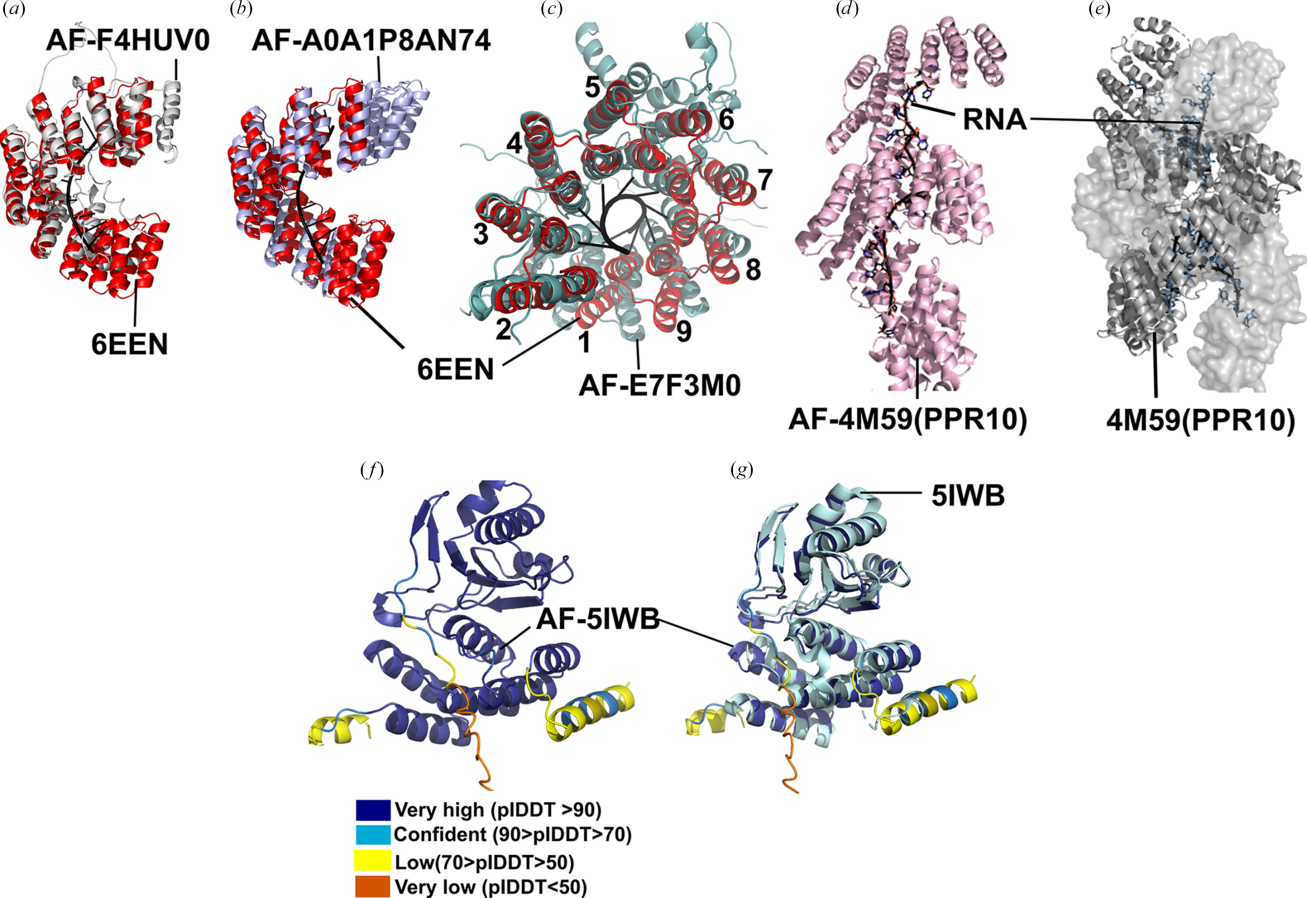
Comparison of *AlphaFold* models with crystal structures. (*a*) Superimposed image of PDB entry 6een and AF-F4HUV0 in grey (AXX17_At1g57940). (*b*) AF-A0A1P8AN74 (PPR1) in blue white and PDB entry 6een in red. (*c*) AF-E7F3M0 (ptcd1) in pale green and PDB entry 6een in red showing nine repeats per superhelical turn. (*d*) Monomeric PPR10 (AF-4M59) shown in pink and RNA shown in black. (*e*) Crystal structure of PPR10 (PDB entry 4m59) with RNA; chain *A* is shown as a cartoon, chain *B* is shown as a surface and two RNA oligonucleotides are shown in black. (*f*) *AlphaFold*-predicted model of AF-5IWB (PLS with MORF) coloured in dark blue, sky blue, yellow and orange based on pLDDT scores. (*g*) Superimposed AF-5IWB and crystal structure of PDB entry 5iwb (PLS with MORF) in pale cyan.

Although *AlphaFold* is good at predicting the helical architecture of these proteins with high confidence, it struggles with predicting the specific superhelical conformation of proteins in the absence of RNA and typically presents them in the compact form with nine repeats per superhelical turn exhibited by protein–RNA complexes (for example PDB entry 6een; Marzano *et al.*, 2024 ▸). Examples of compact conformations of protein-alone predictions are shown in Figs. 7 ▸(*a*), 7 ▸(*b*) and 7 ▸(*c*). Additionally, the *AlphaFold*3 prediction of native PPR10 suggests that the protein could bind RNA in its monomeric form, in agreement with the solution structure studies on PPR10 (Gully, Cowieson *et al.*, 2015 ▸), rather than the crystal structure of PPR10 (PDB entry 4m59; Yin *et al.*, 2013 ▸), resulting in significant implications for the binding of RNA. The *AlphaFold*-predicted PPR10–RNA was placed in the inner groove extending throughout the structure (Figs. 7 ▸*d*), enabling canonical binding, in contrast to the partial canonical and noncanonical binding revealed in the dimeric PPR10 structure (Fig. 7 ▸*d*).

Prediction of PLS-class proteins in complex with MORF proteins also had superhelical architecture with high confidence in the prediction of helical repeats and low confidence in flexible regions and the C-terminal helix (Fig. 7 ▸*f*). Slight inconsistences were observed between all five predicted models: superimposition of the crystal structure of the PLS–MORF complex (PDB entry 5iwb) and AF-5IWB showed consistency in the helical arrangement of P, L and S motifs, interconnected linkers, superhelical architecture and the structure of MORF proteins; however, the C-and N- terminal regions showed variation (Fig. 7 ▸*g*).

Overall, *AlphaFold* reliably modelled the rigid helical arrangement of PPR proteins with high confidence in both protein-only and RNA-bound states, but lacks confidence in the flexible/intrinsically disordered regions, and sometimes the protein alone is shown in a compact conformation rather than a relaxed conformation. *AlphaFold* was less confident in the placement of RNA, often showing RNA bound in the wrong register. As the motif architecture is predicted with high confidence and the RNA is placed in the inner groove, these models can serve as initial models for the fitting of cryo-EM and crystal structures.

## 5. Future directions and outstanding questions

Despite substantial progress, several key questions remain in the PPR protein structural biology field, including:

(i) What is the structure of an intact PPR–DYW editing factor in the absence and presence of RNA?
(ii) What does the structure of a PLS (+MORF) protein bound to RNA look like?
(iii) What does the structure of an S-class repeat protein look like, and does it behave in the same way as a P-class protein?
(iv) What is the conformational diversity of PPR proteins in the absence and presence of RNA?

Although *AlphaFold* predictions generally reproduce the helical architecture of these proteins, inconsistencies among the predicted models from the same sequence, the existence of multiple conformational states that are not all modelled and inaccuracies in RNA positioning all indicate that experimental structure determination is still important to support mechanistic studies. With the possibility of integrative structural biology incorporating solution studies, or cryo-EM methods where families of conformations can be trapped in vitrified ice for imaging, it may be possible to better answer questions about PPR protein dynamics and conformational flexibility, and to clarify functional aspects of RNA editing such as the arrangement of the DYW domain with respect to the RNA target and the role of the gating domain in catalysis. Therefore, future structural studies will likely focus on larger, more complex assemblies and the complementation of crystallo­graphy with computational prediction, cryo-EM, solution scattering and single-molecule techniques.

## Supplementary Material

Supplementary Tables and Figure. DOI: 10.1107/S2053230X26002311/ir5054sup1.pdf

## Figures and Tables

**Table 1:** Summary of selected representative PPR crystal structures, including the PDB accession code, protein subfamily, ligand-binding state and resolution (Å) of the crystal structures reported

PDB code	Protein	Protein subfamily	Binding state	Resolution (Å)
4m57	PPR10	P	Apo	2.86
4m59	PPR10–RNA	P	Bound	2.46
4leu	THA8L	PLS	Apo	2.00
4n2q	THA8–RNA	PLS	Bound	2.80
7a9x	Rmd9–RNA	P	Bound	2.45
4g23	ProRP1	P	Apo	1.80
6bv5	ProRP1–juglone	P	Bound	1.85
4ozs	dPPR10	P	Apo	2.17
6een	dPPR–*atpH*	P	Bound	2.01
5orm	cPPR–Telo1	P	Apo	2.08
5orq	cPPR–DNA	P	Bound	1.95
4wn4	cPPR–polyA	P	Bound	3.85
5i9d	dPPR–U8A2	P	Bound	2.60
5izw	dPLS–PPR	PLS	Apo	1.74
5iwb	dPLS–MORF	PLS	Apo with MORF	1.76
7o4e	OTP86–DYW	PLS	Inactive	2.50
7o4f	OTP86–DYW	PLS	Active	1.65
7w86	DYW1	PLS	Apo	1.80
