# Supplementary material for: Structures of pentatricopeptide repeat proteins
Source: Acta Crystallogr F Struct Biol Commun. 2026 Mar 30;82(Pt 4):101–13. doi: 10.1107/S2053230X26002311 (PMC13041630; doi:10.1107/S2053230X26002311)
Supplement: Supplementary file 1 [file f-82-00101-sup1.pdf]

STRUCTURAL BIOLOGY
COMMUNICATIONS

Volume 82 (2026)

Supporting information for article:

Structures of pentatricopeptide repeat proteins

Anuradha Pullakhandam, Crystal Cooper, Ian D. Small and Charles S. Bond

Table S1 Key Structural Details of PPR Protein Structures in the PDB

Ribosome entries are limited to the examples highlighted in this review.

PDB ID	Protein	Source	Resolution (Å)	Space Group	Unit Cell Lengths (Å), and angles where not 90/120°	R-work/ R-free	Ligands	Year
4G23	PRORP1	*A. thaliana*	1.75	$P\,2_12_12$	41.8, 111.8, 139.8	0.185/ 0.220	Mn^{2+}, Zn^{2+}	2012
4G24	PRORP1	*A. thaliana*	1.75	$P\,2_12_12$	41.8, 112.5, 138.8	0.194/ 0.227	Mn^{2+}, Zn^{2+}	2012
4G25	PRORP1 (SeMet)	*A. thaliana*	1.80	$P\,2_12_12$	41.8, 111.8, 140.2	0.194/ 0.234	Sr^{2+}, Zn^{2+}	2012
4G26	PRORP1	*A. thaliana*	1.85	$P\,2_12_12$	41.8, 111.9, 140.1	0.162/ 0.209	Ca^{2+}, Zn^{2+}	2012
4M57	PPR10	*Z. mays*	2.86	$P\,2_12_12$	68.4, 176.5, 64.5	0.240/ 0.254	None	2013
4M59	PPR10-RNA	*Z. mays*	2.46	$P\,4_3$	83.4, 83.4, 226.9	0.260/ 0.287	*psaJ* RNA	2013
4LEU	THA8L	*A. thaliana*	2.00	*C* 2	117.2, 53.3, 42.3 99.69°	0.204/ 0.253	None	2013
4ME2	THA8	*B. distachyon*	1.60	$P\,6_5$	73.6, 73.6, 61.8	0.212/ 0.244	None	2013
4N2Q	THA8-RNA	*B. distachyon*	2.80	$P\,4_12_12$	90.3, 90.3, 81.7	0.205/ 0.258	Zm4 RNA	2013
4N2S	THA8-RNA	*B. distachyon*	3.00	$P\,4_12_12$	88.3, 88.3, 78.9	0.205/ 0.232	Zm1a-6 RNA	2013
4OE1	PPR10-RNA	*Z. mays*	2.80	$P\,4_3$	83.3, 83.3, 225.5	0.263/ 0.278	*psaJ* RNA	2014
4WN4	cPPR-polyA	Synthetic	3.85	*F* 23	204.7, 204.7, 204.7	0.179/ 0.230	poly(A)	2014

4OZS	dPPR10	Synthetic	2.17	$P\,2_12_12_1$	54.0, 75.0, 85.1	0.217/ 0.265	None	2015
5DIZ	PRORP2	*A. thaliana*	3.20	$P\,1$	69.9, 76.9, 80.1 72.7°, 64.1°, 77.7°	0.228/ 0.272	Zn^{2+}	2016
5I9D	dPPR-U8A2	Synthetic	2.60	$P\,2_12_12_1$	52.6, 84.9, 95.9	0.267/ 0.293	U8A2 RNA	2016
5IZW	dPLS-PPR	Synthetic	1.74	$P\,4_1$	67.4, 67.4, 56.7	0.175/ 0.208	None	2017
5IWB	dPLS-MORF	Synthetic	1.76	$P\,4_12_12$	63.1, 63.1, 136.3	0.179/ 0.199	MORF9	2017
5ORM	cPPR-Telo1	Synthetic	2.08	$P\,2_12_12_1$	86.4, 87.1, 91.5	0.240/ 0.280	None	2018
5ORQ	cPPR-DNA	Synthetic	1.95	$P\,4_12_12$	114.8, 114.8, 83.5	0.189/ 0.228	ssDNA	2018
6BV5	PRORP1-juglone	*A. thaliana*	1.85	$P\,2_12_12$	41.6, 110.8, 140.7	0.196/ 0.223	Juglone (Cys353)	2018
6BV6	PRORP1-juglone	*A. thaliana*	1.90	$P\,2_12_12$	41.7, 112.2, 139.2	0.175/ 0.208	Juglone (multiple)	2018
6BV8	PRORP1-juglone	*A. thaliana*	1.88	$P\,2_12_12$	41.7, 111.3, 139.4	0.200/ 0.236	Mn^{2+}, Juglone	2018
6BV9	PRORP1-juglone	*A. thaliana*	1.92	$P\,2_12_12$	41.8, 111.8, 139.3	0.196/ 0.241	Juglone (extensive)	2018
6EEN	dPPR-atpH	Synthetic	2.01	$P\,1$	43.4, 51.7, 51.9 118.12°, 97.21°, 96.00°	0.183/ 0.248	*atpH* RNA	2019
6XYW	Ribosome	*A. thaliana*	3.86	$C\,1$	Cryo-EM		RNA(2832-MER), RNA(118-MER),RNA (1743-MER)	2020
6LVR	PRORP1-tRNA	*A. thaliana*	2.85	$P\,2_12_12$	84.5, 131.7, 155.6	0.235/ 0.255	tRNA	2020
7A9X	Rmd9-RNA	*S. cerevisiae*	2.45	$P\,3_121$	106.3, 106.3, 128.5	0.199/ 0.233	16-nt RNA	2021

7A9W	Rmd9-RNA	*S. cerevisiae*	2.55	*P* 3_121	106.7, 106.7, 128.2	0.192/ 0.233	2-nt RNA	2021
7O4E	OTP86-DYW	*A. thaliana*	2.50	*C* 2	124.6, 30.6, 77.5 β =125.8°	0.222/ 0.271	Inactive	2021
7O4F	OTP86-DYW	*A. thaliana*	1.65	*P*2_12_12	117.6, 132.9, 30.6	0.221/ 0.268	Active	2021
7PUA	Ribosome	*Trypanosoma brucei brucei*	3.60	*C* 1	Cryo-EM		9S rRNA (chain A), 9S rRNA (chain B) Dihydroflavine-Adenine Dinucleotide Adenosine-5'-Triphosphate S-(2-([N-(2-Hydroxy-4-([Hydroxy (Oxido)Phosphino]Oxy)-3,3-Dimethylbutanoyl)-Beta-Alanyl]Amino)Ethyl) Decanethioate, Guanosine-5'-Diphosphate, Phosphate Ion, Zinc Ion, Magnesium Ion	2022
7W86	DYW1	*A. thaliana*	1.80	*P* 2_1	32.9, 45.2, 37.0 β=98.0	0.177/ 0.234	None	2023
8ANY	Ribosome	*Homo sapiens*	2.85	*C* 1	Cryo-EM		12S mitochondrial rRNA, A/A-tRNA, P/P-tRNA, E/E-tRNA, 16S mitochondrial rRNA, mitochondrial tRNAVal, mRNA, Nicotinamide-Adenine-dinucleotide, Adenosine-5'-Triphosphate, Guanosine-5'-Diphosphate, Spermine, FE2/S2 (inorganic)cluster, Spermidine, Valine, 1,4-Diaminobutane,	2023

							Zinc ion, Potassium ion, Magnesium ion.	

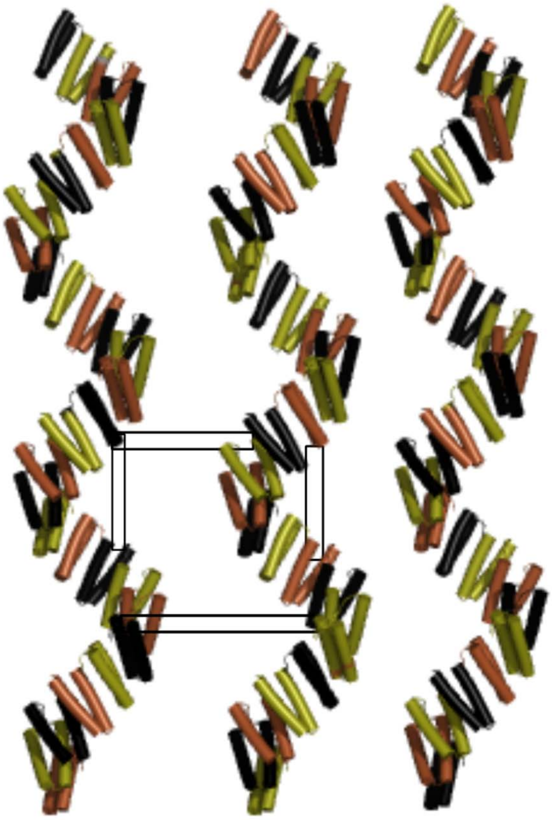

Figure S1 Helical disorder in PPR proteins. Superhelical structure of synthetic P-class PPR protein with 3.5 motifs (PDB4OZS) motifs are coloured in brown, deep olive and black triplets. Rotational and translational shifts along the superhelical axis allow a brown motif to occupy any of the three positions, resulting in an averaged superhelical structure (adapted from (Gully, Shah*, et al.*, 2015)).

Table S2 PPR structures of different organisms retrieved from AlphaFold database, with their model Id, gene, UniProt accession, organism and global metric value.

Model Entity Id	Gene	Uniport Accession	Organism	Global Metric Value
AF-A0A1P8AN74-F1	PPR1	A0A1P8AN74	*Arabidopsis thaliana*	87.81
AF-A0A1I9LRQ7-F1	PPR3	A0A1I9LRQ7	*Arabidopsis thaliana*	85.5
AF-A0A1I9LM09-F1	PPR4	A0A1I9LM09	*Arabidopsis thaliana*	82.69
AF-K7SUA7-F1	PPR	K7SUA7	*Arabidopsis thaliana*	76.19
AF-B6VG96-F1	At3g22690	B6VG96	*Arabidopsis thaliana*	95.38
AF-A0A1P8ARL9-F1	At1g12615	A0A1P8ARL9	*Arabidopsis thaliana*	77.5
AF-F4JUM8-F1	DL3140C	F4JUM8	*Arabidopsis thaliana*	48.06
AF-F4K742-F1	At5g28420	F4K742	*Arabidopsis thaliana*	93.69
AF-A0A1I9LRG4-F1	At3g50420	A0A1I9LRG4	*Arabidopsis thaliana*	77
AF-A0A5S9XJ59-F1	C24_LOCUS15137	A0A5S9XJ59	*Arabidopsis thaliana*	62.59
AF-A0A1I9LQL2-F1	At3g13770	A0A1I9LQL2	*Arabidopsis thaliana*	86.75
AF-A0A1P8AS46-F1	At1g53330	A0A1P8AS46	*Arabidopsis thaliana*	87.38
AF-Q8VYZ8-F1	At1g64430	Q8VYZ8	*Arabidopsis thaliana*	72.19
AF-A0A1P8B0F9-F1	AHG11	A0A1P8B0F9	*Arabidopsis thaliana*	88.5

Model Entity Id	Gene	Uniport Accession	Organism	Global Metric Value
AF-A0A5S9WRX3-F1	AN1_LOCUS5229	A0A5S9WRX3	*Arabidopsis thaliana*	82.12
AF-Q9CAY8-F1	F24K9.2	Q9CAY8	*Arabidopsis thaliana*	61.78
AF-Q9M2A8-F1	T12K4_10	Q9M2A8	*Arabidopsis thaliana*	53.03
AF-A0A1P8B0G9-F1	AHG11	A0A1P8B0G9	*Arabidopsis thaliana*	88.56
AF-F4J555-F1	At3g17370	F4J555	*Arabidopsis thaliana*	79.06
AF-A0A1P8B0J3-F1	AHG11	A0A1P8B0J3	*Arabidopsis thaliana*	87.56
AF-A0A1I9LQP8-F1	MEF12	A0A1I9LQP8	*Arabidopsis thaliana*	80.5
AF-A0A178URB5-F1	AXX17_At5g10340	A0A178URB5	*Arabidopsis thaliana*	67.88
AF-F4I5L3-F1	At1g77150	F4I5L3	*Arabidopsis thaliana*	78.56
AF-A0A178WJA5-F1	AXX17_At1g39080	A0A178WJA5	*Arabidopsis thaliana*	73
AF-A0A1P8AWU6-F1	At1g11710	A0A1P8AWU6	*Arabidopsis thaliana*	75.25
AF-A0A1P8B5Z8-F1	ABO8	A0A1P8B5Z8	*Arabidopsis thaliana*	78.88
AF-F4HUV0-F1	At1g28000	F4HUV0	*Arabidopsis thaliana*	73.94

Model Entity Id	Gene	Uniport Accession	Organism	Global Metric Value
AF-A0A178WJJ1-F1	AXX17_At1g57940	A0A178WJJ1	*Arabidopsis thaliana*	69.94
AF-F4I704-F1	At1g77340	F4I704	*Arabidopsis thaliana*	90.31
AF-Q1G3C0-F1	At2g27229	Q1G3C0	*Arabidopsis thaliana*	39.31
AF-A0A1I9LM77-F1	At3g59300	A0A1I9LM77	*Arabidopsis thaliana*	65.12
AF-F4K6R8-F1	At5g14350	F4K6R8	*Arabidopsis thaliana*	48.41
AF-A0A1P8B7C5-F1	At4g21880	A0A1P8B7C5	*Arabidopsis thaliana*	65.25
AF-A0A1P8B0G8-F1	AHG11	A0A1P8B0G8	*Arabidopsis thaliana*	88.12
AF-A0A1I9LSM9-F1	At3g46870	A0A1I9LSM9	*Arabidopsis thaliana*	72
AF-A0A178W9C4-F1	AXX17_At1g13100	A0A178W9C4	*Arabidopsis thaliana*	93.56
AF-A0A654FE62-F1	AN1_LOCUS15259	A0A654FE62	*Arabidopsis thaliana*	62.62
AF-A0A178UMH8-F1	At5g24060	A0A178UMH8	*Arabidopsis thaliana*	61.06
AF-A0A1I9LPC7-F1	At3g04760	A0A1I9LPC7	*Arabidopsis thaliana*	76.75
AF-Q949V7-F1	At3g59300	Q949V7	*Arabidopsis thaliana*	61.44

Model Entity Id	Gene	Uniport Accession	Organism	Global Metric Value
AF-A0A1P8B6V5-F1	At4g26800	A0A1P8B6V5	*Arabidopsis thaliana*	88.12
AF-A0A178W811-F1	AXX17_At1g14620	A0A178W811	*Arabidopsis thaliana*	79.62
AF-F4I8W7-F1	At1g11470	F4I8W7	*Arabidopsis thaliana*	70.94
AF-A0A178WHP1-F1	AXX17_At1g57940	A0A178WHP1	*Arabidopsis thaliana*	70.88
AF-A0A1I9LM78-F1	At3g59300	A0A1I9LM78	*Arabidopsis thaliana*	63.81
AF-A0A1P8AST8-F1	At1g62350	A0A1P8AST8	*Arabidopsis thaliana*	80.69
AF-A0A1P8AQY1-F1	At1g64430	A0A1P8AQY1	*Arabidopsis thaliana*	67.19
AF-A0A1P8B4N6-F1	GRS1	A0A1P8B4N6	*Arabidopsis thaliana*	82.5
AF-F4I221-F1	At1g63320	F4I221	*Arabidopsis thaliana*	91.31
AF-F4HV54-F1	At1g47940	F4HV54	*Arabidopsis thaliana*	36
AF-F4IDY2-F1	At1g18900	F4IDY2	*Arabidopsis thaliana*	64.81
AF-Q9CAY6-F1	F24K9.4	Q9CAY6	*Arabidopsis thaliana*	78.44
AF-A0A178WKE5-F1	AXX17_At1g56780	A0A178WKE5	*Arabidopsis thaliana*	90.19

Model Entity Id	Gene	Uniport Accession	Organism	Global Metric Value
AF-A0A1P8ASR0-F1	At1g62350	A0A1P8ASR0	*Arabidopsis thaliana*	86.62
AF-A0A1P8BA34-F1	MEE6.24	A0A1P8BA34	*Arabidopsis thaliana*	88.88
AF-A0A1P8BH16-F1	MPO12.110	A0A1P8BH16	*Arabidopsis thaliana*	76.56
AF-F4KFP7-F1	MZF18.6	F4KFP7	*Arabidopsis thaliana*	62.5
AF-A0A1P8B6L5-F1	MEF32	A0A1P8B6L5	*Arabidopsis thaliana*	82.38
AF-A0A1P8B7P3-F1	MEF35	A0A1P8B7P3	*Arabidopsis thaliana*	85.31
AF-A0A1P8BF21-F1	MDH9.15	A0A1P8BF21	*Arabidopsis thaliana*	74.56
AF-A0A1P8BA16-F1	MEE6.24	A0A1P8BA16	*Arabidopsis thaliana*	83.12
AF-F4KFP8-F1	MZF18.6	F4KFP8	*Arabidopsis thaliana*	63.06
AF-A0A1P8BHN8-F1	MLN1.16	A0A1P8BHN8	*Arabidopsis thaliana*	90.31
AF-A0A1P8AN66-F1	EMB3103	A0A1P8AN66	*Arabidopsis thaliana*	79.69
AF-A0AAA9T105-F1	PTCD1	A0AAA9T105	*Bos taurus*	62.72
AF-A0AAA9TCR3-F1	PTCD2	A0AAA9TCR3	*Bos taurus*	84.69

Model Entity Id	Gene	Uniport Accession	Organism	Global Metric Value
AF-A0AAA9TK67-F1	PTCD1	A0AAA9TK67	*Bos taurus*	62.41
AF-E1BNM9-F1	PTCD1	E1BNM9	*Bos taurus*	63.34
AF-A0AAA9SQN1-F1	PTCD1	A0AAA9SQN1	*Bos taurus*	64.69
AF-A0AAA9SV15-F1	PTCD2	A0AAA9SV15	*Bos taurus*	70.06
AF-A0A3Q1MQ62-F1	PTCD1	A0A3Q1MQ62	*Bos taurus*	60
AF-A0AAA9TYF6-F1	PTCD1	A0AAA9TYF6	*Bos taurus*	59.12
AF-E7F3M0-F1	ptcd1	E7F3M0	*Danio rerio*	68.5
AF-A0A2R8RTA8-F1	ptcd1	A0A2R8RTA8	*Danio rerio*	70.75
